# Diverse musculature layers in three species of octopus support precise motor control yet lack smooth muscle

**DOI:** 10.1098/rsos.250684

**Published:** 2025-09-10

**Authors:** Sarah L. West, Trevor J. Wardill

**Affiliations:** ^1^ Department of Ecology, Evolution, and Behavior, University of Minnesota, Saint Paul, MN, USA

**Keywords:** octopus, muscle, arm control, fluorescence microscopy, scanning electron microscopy, cephalopod, morphology, smooth muscle

## Abstract

Octopus intrinsic arm musculature is often referred to as entirely obliquely striated muscle. However, only three muscle layers have been systematically shown as striated muscle. Because molluscan muscle control can vary greatly (i.e. smooth versus striated types), a systematic examination of each arm muscle layer is necessary to understand its neuromuscular control. Here, we use two-photon microscopy to determine if striations are present in the eight layers and trabeculae of intrinsic arm muscle in *Octopus bimaculoides* (California two-spot octopus) and *Abdopus aculeatus* (‘prickly octopus’). We also use scanning electron microscopy to examine the arm of *Octopus bocki* (Bock’s pygmy octopus). We confirm for the first time, to our knowledge, that each intrinsic arm muscle layer from multiple octopus species is obliquely striated. Furthermore, we find that the two layers of longitudinal muscle—divided by the median oblique layer—exhibit different morphology. This occurs in all three species examined, with significantly lower muscle fibre density in the internal longitudinal layer of *O. bimaculoides* and *A. aculeatus*, and smaller internal mitochondria cores (with larger muscle area) compared with the external longitudinal layer in *O. bocki*. This suggests additional functional muscle layers that would give octopuses even greater precision in motor control of their arms.

## 1. Introduction

The neural control of octopus arms is beginning to be deciphered using ultrastructure and molecular atlas approaches [1–4]. Each arm maintains a constant volume, but bends, twists, shortens and elongates as the intrinsic arm muscles contract along various planes [5] and at any point along the arm [6]. This, along with the hundreds of independently directionally controllable suckers that line the oral surface of each arm, creates an immense number of degrees of freedom that must be controlled to make successful arm and sucker goal-directed movements. This motor control conundrum regarding how the octopus nervous system coordinates its eight arms is a question of interest to neuroscientists and roboticists [7,8].

**Figure 1.**
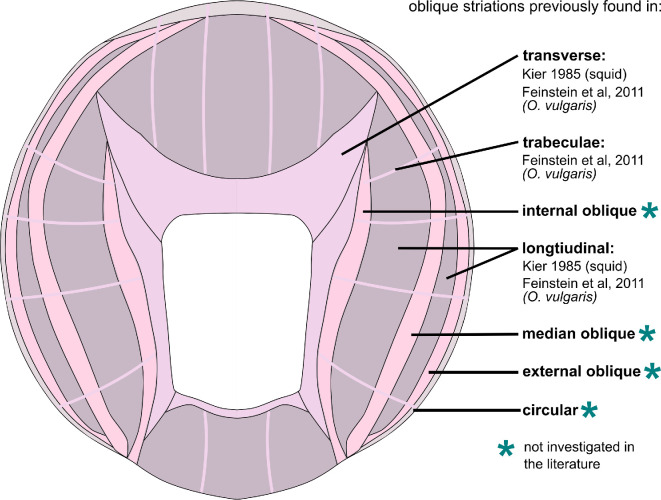
Diagram representation of the intrinsic muscle layers of the octopus arm. The transverse layer, the trabeculae and the longitudinal layers have been found to be obliquely striated in two squid species, *Loligo pealei* and *Illex illecebrosus* [12], as well as in *Octopus vulgaris* [13]. The muscle types of the internal, median and external oblique layers, as well as the circular layer, have not been previously investigated in the literature in any cephalopod species [2,13].

The composition of a muscle group within an arm can also greatly influence its motor control strategy. For example, the anterior byssus retractor muscle (ABRM) of *Mytilus*, a molluscan bivalve, exhibits a phenomenon called a catch mechanism (for review, see [9,10]). The ABRM holds the two halves of the bivalve’s shell closed and is partially composed of smooth, unstriated muscle fibres. These smooth fibres can use what is called a catch mechanism, which allows for sustained contraction of the muscle without continuous neural input and without expending additional energy, and is unique to smooth muscle of some invertebrate species. A recent study [11] found evidence that cuttlefish use a catch mechanism in the control of the skin papillae, which raises the possibility that this phenomenon may be present among molluscan cephalopods. However, there have been no other reports of the catch mechanism occurring in cephalopods. If present in the intrinsic muscle of the arm, smooth muscle with the capability to perform the catch mechanism would add additional nervous system control options of the arm, such as providing a temporary rigid substrate (e.g. smooth muscles in catch state) to enhance leverage from striated muscles. Therefore, it is important to understand the type and composition of each muscle group that assists with arm movement. The intrinsic arm musculature is made up of seven muscular layers running in the transverse, longitudinal, oblique and circular directions relative to the longitudinal axis of the arm, as well as the trabeculae of the transverse muscle (figure 1; [14]). Across the literature, it has been generalized that each of these muscle groups is obliquely striated in all cephalopods [15]. However, to the best of our knowledge, this has not been systematically investigated in each muscle group in an octopus species nor documented in the literature.

Studies by Socastro [16] and Gonzalez-Santander & Socastro Garcia-Blanco [17] explored the microstructure of the arm muscle using electron microscopy in *Sepia officinalis* (cuttlefish), *Sepiola rondeletii* (bobtail squid) and two octopus species: *Octopus vulgaris* (common octopus) and *Eledone cirrhosa* (curled octopus; [16,17]). These studies confirmed that the arm muscle generally contained obliquely striated muscle and put an end to debates around 1970 over whether such invertebrate muscles were striated [18]. However, each of the arm muscle layers was not systematically surveyed across species and muscle groups, and the ultrastructure of each muscle layer was not confirmed. More recently, smooth-like muscle fibres have been reported in cuttlefish tentacles, which have a similar structure to the arms, during development [19,20]. Therefore, it is important to investigate each muscle group of the arm to determine if it consists of striated or smooth muscle fibres.

Only a handful of works have investigated the ultrastructure of specific muscle groups in the cephalopod arm. Notably, Kier in 1985 performed a systematic study of the transverse muscle of the arm of two squid species, *Loligo pealei* and *Illex illecebrosus,* using electron microscopy [12]. Longitudinal muscle fibres near the transverse muscle were observed and thought to probably be obliquely striated, but were not specifically investigated [12]. Since that time, there has been very little evidence in the literature supporting that all the arm muscle groups are also obliquely striated, in squid or other cephalopods, as Kier’s later works focus on the comparison between the obliquely striated transverse muscle in the squid arm versus the cross-striated muscle in the transverse muscle of the tentacle [21,22]. A more recent study performing electron microscopy in the arm of *O. vulgaris* incidentally shows oblique striations in the transverse and longitudinal muscles [13]. However, this leaves many of the muscle groups unreported in the literature, including the three layers of oblique muscle and the circular layer, and no documented systematic investigation of the trabeculae or longitudinal muscles (figure 1). The ultrastructure of each independent muscle group may very well be different, as the physiology and biomechanics vary between them, suggesting different functional roles [6,23]. Therefore, it is important to investigate each muscle layer with modern techniques to determine if each is composed of smooth or striated muscle fibres.

Here, we investigated the structure of each layer of the intrinsic muscle of the arm in three octopus species: *Octopus bimaculoides* (the California two-spot octopus), *Abdopus aculeatus* (the ‘prickly’ or ‘algae’ octopus) and *Octopus bocki* (Bock’s pygmy octopus). We selected these highly divergent species (in anatomy, behaviour and habitat use) to determine if general muscle types and arrangements were conserved. Using actin staining with two-photon fluorescence imaging and scanning electron microscopy (SEM), we confirm for the first time, to our knowledge, that all seven muscle groups are composed of obliquely striated muscle fibres and that no smooth muscle fibres were observed (indicating catch muscles are not likely present). While we did sample arm base tissue for *O. bimaculoides* and *A. aculeatus*, and tissue near the tip for *O. bocki*, the main muscle groups have previously been shown to remain in the same proportions from base to tip for *O. bimaculoides* [14]. In addition, we find that the internal layer of the longitudinal muscle in *O. bimaculoides* and *A. aculeatus* is less densely packed with cell fibres than the external layer. Additionally, we observed larger muscle cores (i.e. containing mitochondria) and relatively smaller cell cross-sectional areas for the external versus internal longitudinal muscle layers for *O. bocki*. This suggests possible differences in the biomechanics of different portions of the longitudinal muscle, which have not been described before.

## 2. Results

**Figure 2.**
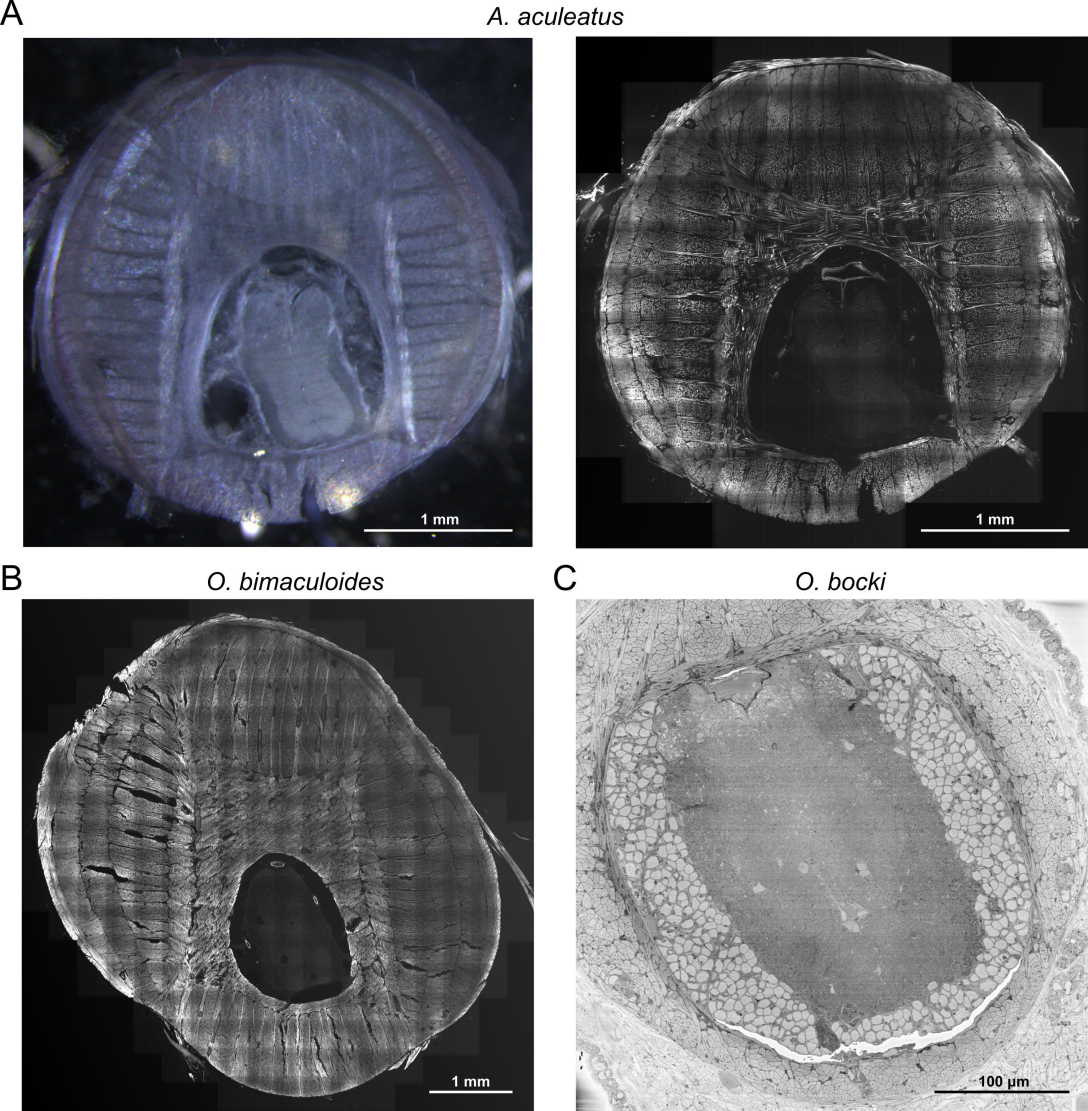
(A) (Left) Vibratome cross section of *Abdopus aculeatus* arm (individual no.1, male, arm L2; 100 μm thickness), stained with AlexaFluor 633 phalloidin and imaged with a stereomicroscope (Leica M165FC, PlanApo 1× objective, 1.5× zoom). (Right) The same muscle slice was imaged with a two-photon fluorescence microscope (Bruker Ultima IV *in vivo*, Carl Zeiss 25×objective, 1× zoom). (B) Cross section of *Octopus bimaculoides* arm (individual no.1, female, arm R2), stained with AlexaFluor 633 and imaged with a two-photon fluorescence microscope (as above). (C) Cross section of distal *Octopus bocki* arm (individual no.1, female, arm L1) imaged with a scanning electron microscope (Teneo Volumescope system).

We conducted two-photon imaging in cross-sectional slices taken from both the R2 and L2 arms in two *O. bimaculoides* and two *A. aculeatus* individuals (figure 2A–C). Slices were stained with AlexaFluor 633 phalloidin, an F-actin fluorescent probe that binds the myofilaments within muscle fibres (Life Technologies, catalogue no. A22284). The different muscle groups are visible under AlexaFluor 633 staining from a wide field of view (figure 2A, B). Note that the structural anatomy labelled in figure 1 applies to each of the three species used in this study. Notably, *A. aculeatus* shows sparser muscle fibre labelling than *O. bimaculoides*, most evident in the transverse muscle and the internal layer of longitudinal muscle, suggesting the muscle is less closely packed in *A. aculeatus*. The arms of *A. aculeatus* no. 2 have extreme muscle fibre sparseness compared with those of even *A. aculeatus* no. 1, although the muscle layer thickness appears proportional to the width of the arm overall (electronic supplementary material, figure S1). This could be owing to this animal’s juvenile status or owing to atrophy of the arm muscles that may have been a result of ill health before the animal’s euthanasia. SEM imaging was provided by Neascu & Crook [24] and performed in the distal tips of the arms of two *O. bocki* individuals (figure 2D; [24]). All muscle layers are visible in each animal (figure 2D; electronic supplementary material, figure S2).

### 2.1. Striations are present in all muscle groups of *Octopus bimaculoides* and *Abdopus aculeatus*

**Figure 3.**
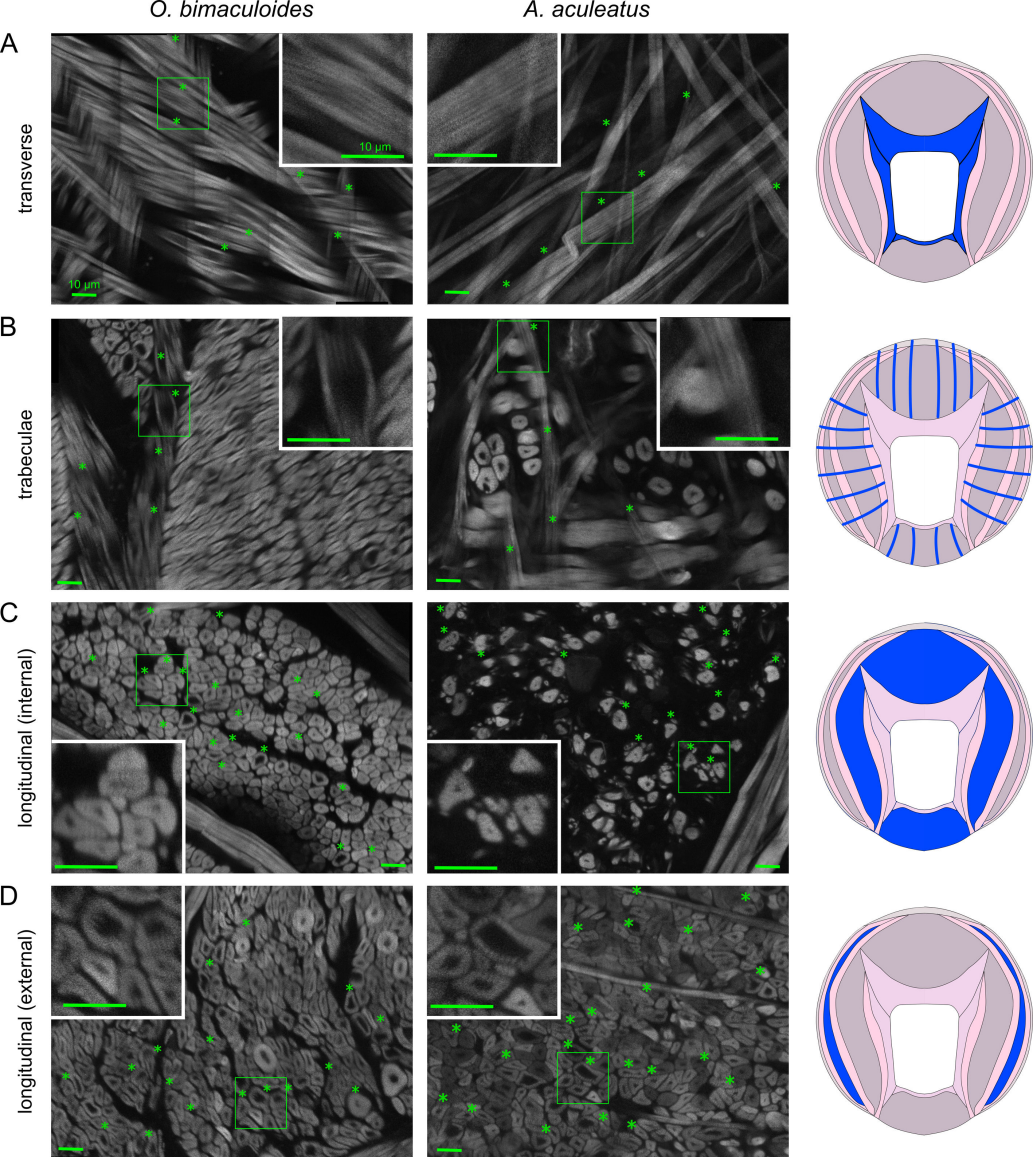
(A–D) Examples of muscle fibre striations in transverse muscle, trabeculae and the internal and external layers of longitudinal muscle in *O. bimaculoides* (left) and *A. aculeatus* (right). Tissue sections were stained with AlexaFluor 633 phalloidin and images collected with a two-photon fluorescence microscope (Bruker Ultima IV *in vivo***,** Carl Zeiss 25× objective, 10× zoom). Stars indicate individual instances of visible striations within the specified muscle group, while insets show an additional 2.5× zoom view of striation examples. All scale bars 10 μm. Source of each image: female *O. bimaculoid*es: (A): individual no. 1, arm R2; (B): no. 1, R2; (C): no. 2, R2; (D): no. 2, R2; male *A. aculeatus*: (A): no. 1, R2; (B): no. 2, R2; (C): no. 1, R2; (D): no. 1, R2. Proximal arm sections were made after sucker rows 8–11.

**Figure 4.**
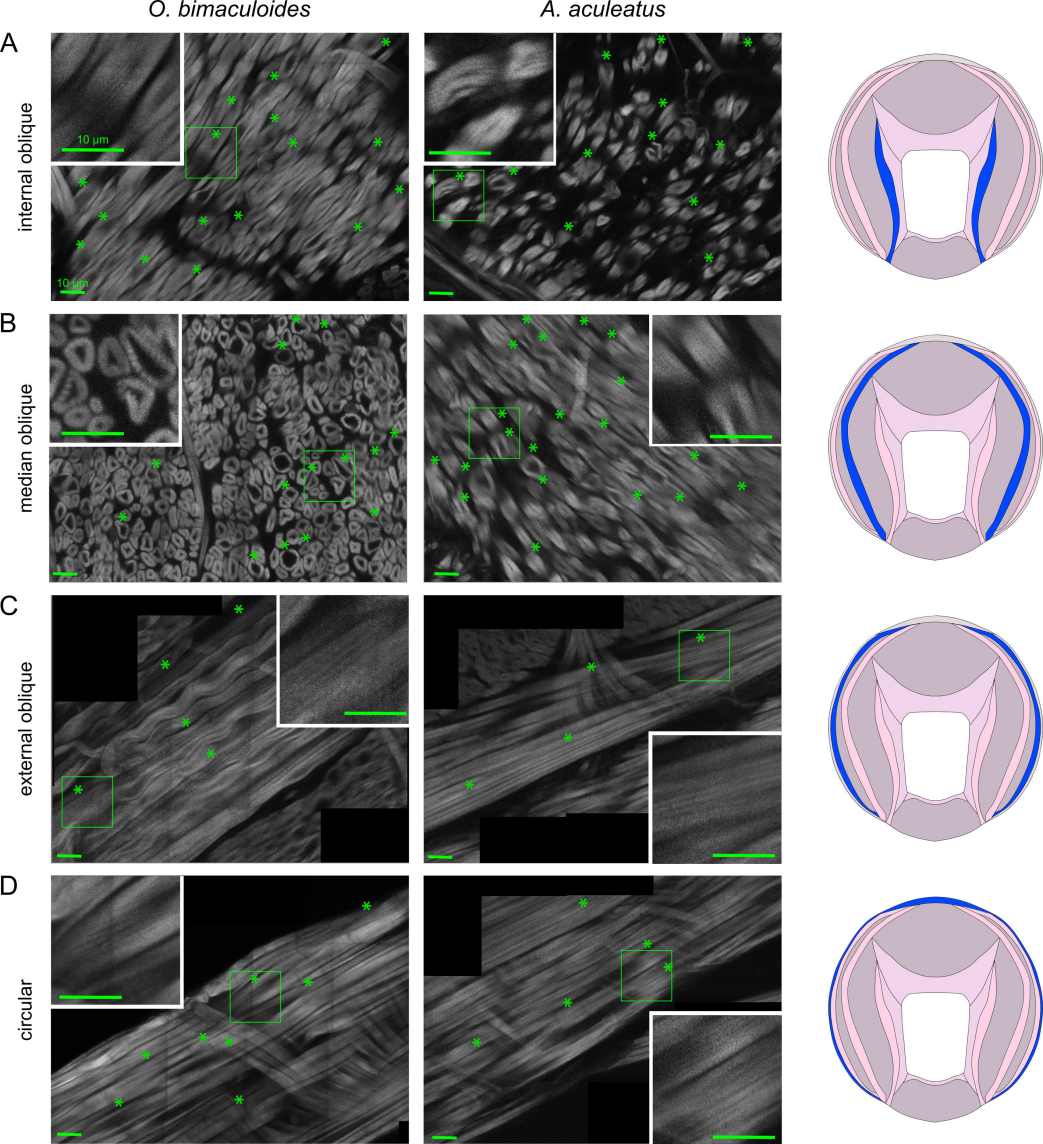
(A–D) Examples of muscle fibre striations in internal, median and external oblique muscles as well as the circular muscle in *O. bimaculoides* (left) and *A. aculeatus* (right). Tissue sections were stained with AlexaFluor 633 phalloidin and images were collected with a two-photon fluorescence microscope (Bruker Ultima IV *in vivo***,** Carl Zeiss 25× objective, 10× zoom). Stars indicate individual instances of visible striations within the specified muscle group, while insets show an additional 2.5× zoom view of striation examples. All scale bars 10 μm. Source of each image: female *O. bimaculoid*es: (A): individual no. 2, arm R2; (B): no. 1, R2; (C): no. 1, R2; (D): no. 2, R2; male *A. aculeatus*: (A): no. 1, R2; (B): no. 1, R2; (C): no. 1, L2; (D): no. 1, L2. Proximal arm sections were made after sucker rows 8–11.

Striations were detected in all muscle groups of both *O. bimaculoides* and *A. aculeatus*, in all individuals and in both arms (figures 3 and 4). The only area where striations were not explicitly recorded was the internal layer of longitudinal muscle in L2 of *A. aculeatus* individual no. 2, owing to the very sparse presence of internal longitudinal muscle fibres in this animal (electronic supplementary material, figure S1). Striations in the transverse muscle have been previously reported in other cephalopod species [12,13], and we confirm this finding here (figure 3A). Striations are also present in the transverse muscle of *A. aculeatus* (figure 3A), and in the trabeculae of both species (figure 3B), which are extensions of the transverse muscle [15]. The longitudinal muscle in *O. vulgaris* has been reported as striated [13], and we show in *O. bimaculoides* and *A. aculeatus* that both the internal and external layers of longitudinal muscle are striated (figure 3C,D)*.* Finally, striations are present in the internal, median and external oblique muscle layers as well as in the circular muscle that circumnavigates the periphery of the intrinsic arm (figure 4)*.*

### 2.2. Striations are present in all muscle groups of *Octopus bocki*

**Figure 5.**
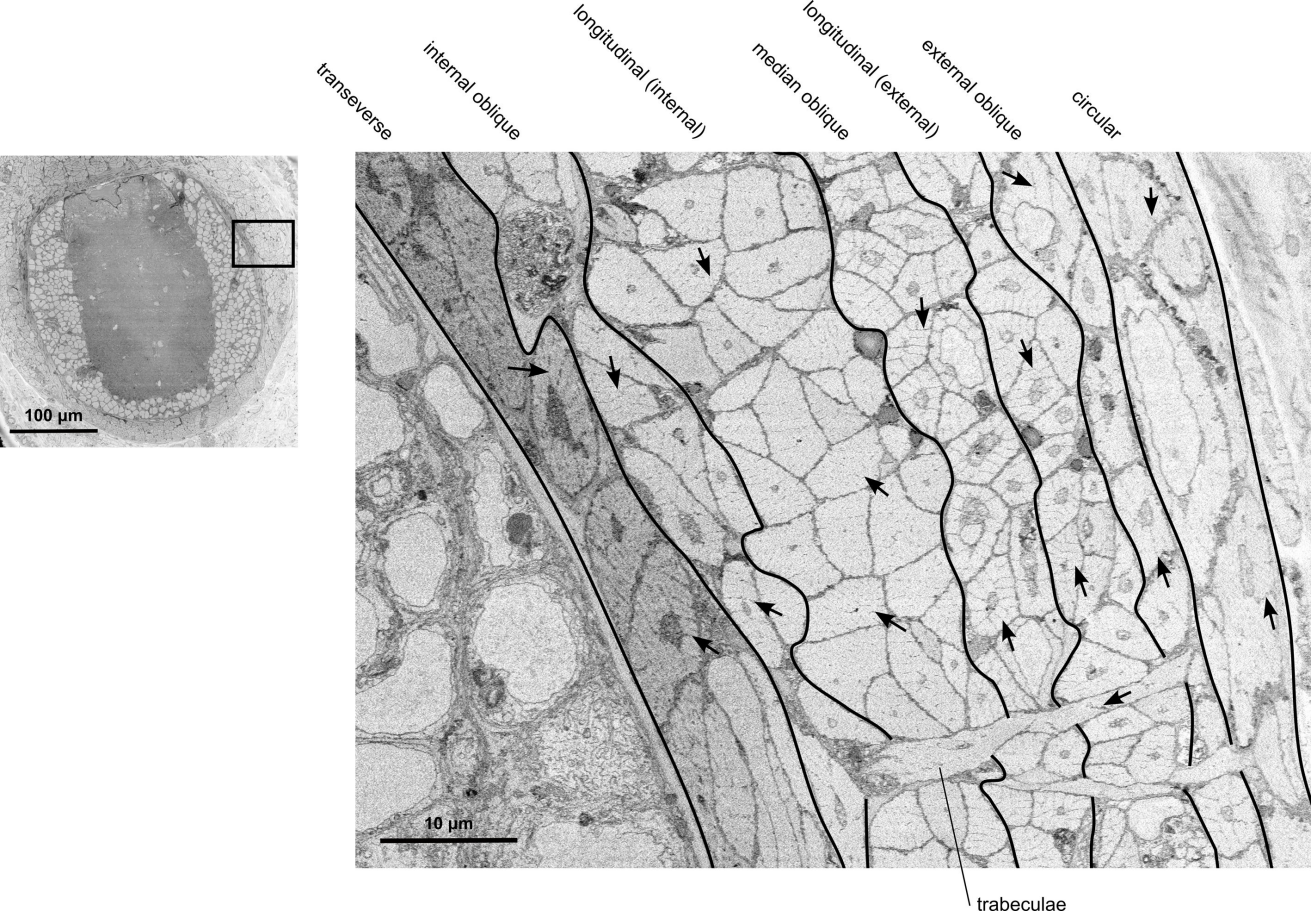
Scanning electron microscopy image of the distal tip of the arm of *O. bocki* (individual no. 1, arm L1) showing each muscle layer. Arrows indicate examples of striations. Inset scale bar 10 μm.

Data from a third species, *O. bocki*, were provided from a separate study by Neacsu & Crook [24]. SEM was conducted on the distal tips of arms from two sub-adult individuals (figure 2C). In individual no. 1, muscle fibre striations are visible in all layers of the intrinsic arm muscles (figure 5). Imaging was performed in individual no. 2 at a lower resolution (see Methods), making the detection of striations more difficult. However, striations are visible in the median oblique, external longitudinal and external oblique layers of individual no. 2 (electronic supplementary material, figure S2). Together, our results show that each layer of the intrinsic arm muscle is striated in three octopus species: *O. bimaculoides*, *A. aculeatus* and *O. bocki*.

### 2.3. Muscle fibre density of internal and external longitudinal muscle

**Figure 6.**
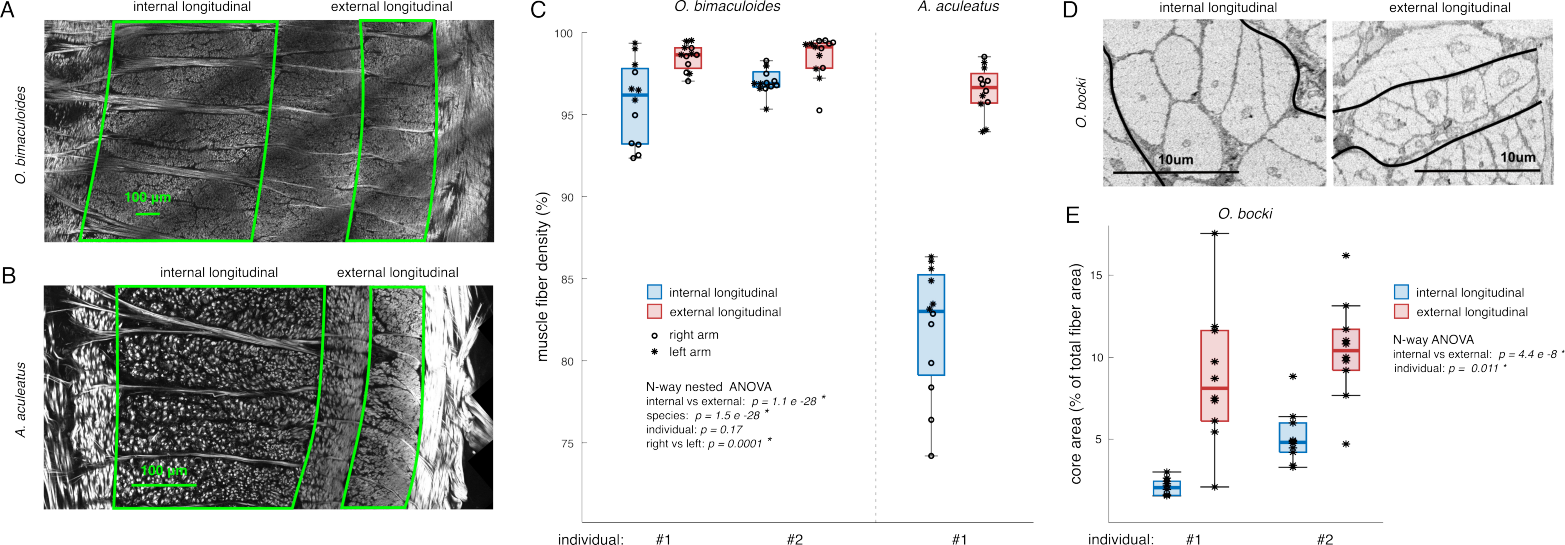
(A,B) Example images from *O. bimaculoides* (individual no. 1, arm L2) and *A. aculeatus* (no. 1, (*l*)) illustrating the lower muscle fibre density of the internal longitudinal muscle layer compared with the external longitudinal layer in both species. Tissue sections were stained with AlexaFluor 633 phalloidin and images collected with a two-photon fluorescence microscope (Bruker Ultima IV *in vivo*, Carl Zeiss 25× objective, 6A: 10× zoom, 6B 3× zoom). (C) Quantification of muscle fibre density in the internal and external longitudinal muscle layers in the three adult specimens. (D) Muscle fibres of the internal and external longitudinal layers of *O. bocki* (no. 1, (*l*)); note the differences in cell morphology. (E) Quantification of the cross-sectional area of mitochondrial core relative to the muscle fibre area (%) for internal and external longitudinal muscles in two adult *O. bocki* specimens.

During imaging, we noticed a possible difference in muscle fibre density of the longitudinal muscle between *O. bimaculoides* and *A. aculeatus,* as well as between the internal and external longitudinal layers within each species. *Abdopus aculeatus* appears markedly more sparse in all layers compared with *O. bimaculoides*, but particularly in the internal longitudinal layer (figure 6A,B). In both species, the internal longitudinal layer appears sparser than the external longitudinal layer (figure 6A,B). To investigate the differences between the layers of longitudinal muscle, the density of muscle fibres in the internal and external longitudinal layers was digitally quantified in each of the adult specimens (*O. bimaculoides* no. 1 and 2, *A. aculeatus* no. 1; figure 6C; see Methods). As hypothesized, external longitudinal muscle has increased muscle fibre density over internal (figure 6C; *p* = 1.1 × 10^−28^, d.f. = 1), while *A. aculeatus* has lower muscle fibre density than *O. bimaculoides* (*p* = 1.5 × 10^−28^, d.f. = 1). The left arm was found to have significantly higher fibre density than the right (*p* = 0.0001, d.f. = 1); however, this is probably owing to the effects of each arm resulting in different muscle slices and a relatively low sample size. The effect of individuals is not significant (*p* = 0.17, d.f. = 1).

### 2.4. Relative muscle and mitochondrial core areas of internal and external longitudinal muscle

While the thin layers and the nature of the SEM images make the density of these layers difficult to assess in *O. bocki*, visual inspection shows morphological differences in the muscle fibres of the internal and external longitudinal layers (figure 6D). The internal fibres have larger areas and smaller mitochondrial cores than the external fibres, further supporting that there may be structural differences between these two layers across octopus species. Thus, we quantified their cross-sectional areas (*O. bocki* individuals no. 1 and no. 2), measuring 10 external and internal longitudinal muscles (from the same sections shown in figure 5; electronic supplementary material, figure S2; also see Methods). We found that internal fibres have significantly smaller mitochondrial cores (0.70 ± 0.33 μm^2^) and larger muscle cross-sectional area (20.12 ± 4.66 μm^2^) than the external fibres (1.27 ± 0.57 μm^2^ and 13.10 ± 3.08 μm^2^, respectively), as well as the relative area of the core was smaller for internal longitudinal muscles (3.64 ± 1.94% for internal, 9.63 ± 3.69% for external; muscle fibre area: *p* = 1.01 × 10^−6^, core area: *p* = 2.93 × 10^−4^, core relative area (%): *p* = 4.4 × 10^−8^, d.f. = 1; figure 6E). The effect of individuals was also significant in the case of muscle fibre area (*p* = 0.040, d.f. = 1) and core relative area (*p* = 0.0112, d.f. = 1), but not in core area (*p* = 0.068, d.f. = 1).

## 3. Discussion

In the present study, we used fluorescent actin staining with two-photon fluorescence imaging or SEM to observe the composition of each intrinsic muscle layer of the arm across three octopus species: *O. bimaculoides*, *A. aculeatus* and *O. bocki*. We confirmed that each muscle layer in all three species is obliquely striated. Moreover, we found that in *O. bimaculoides* and *A. aculeatus*, the internal layer of longitudinal muscle is less dense with muscle cell fibres than the external longitudinal layer, suggesting possible physiological or biomechanical differences between the two layers that have not been described before. Studies to understand the neural control of these muscles have elucidated some possible routes for functional coordination [4,23–26], yet considerable work remains to understand their dynamic control as well as neural links to suckers and sensory information (local and central).

To the best of our knowledge, no published literature has previously confirmed the presence of obliquely striated fibres in all muscle layers of the octopus intrinsic arm. Our findings rule out the possibility that some arm muscles can be held stiffened via the catch mechanism found in the smooth muscle of other molluscan species [10], allowing other muscles to insert and exert force on a rigid substrate. Instead, each muscle fibre along the length of the arm must be controlled with continuous action potential input from the central and peripheral nervous system. While octopuses were not found to have catch-like muscles in their arms, such a ‘lockable’ control system mixed with linear controlled muscles could be used to aid soft robotics designs.

Apart from a catch mechanism, the presence of gap junctions that electrically couple adjacent muscle fibres may also provide a method to simplify neural control of the arms. In this case, action potentials delivered to one fibre could also activate adjacent fibres, relieving the need for continuous input to each. Gap junctions are featured in a variety of physiological systems across a wide range of taxa [27,28] and have been found between fibres of obliquely striated muscle in cephalopods [29], including in the chromatophore radial muscle in the squid *Loligo opalescens* [30,31], in the mantle of *Alloteuthis* and *Sepia* [29], and the walls of the digestive tract of *Sepia officinalis* [32]. A handful of studies have reported that the arm muscle of cephalopods lacks myo-muscular junctions [6,13,33]; however, these studies used electron microscopy to visually identify junctions between fibres as an aside to larger anatomic investigations. Future experiments using dye tracers that diffuse through gap junctions, such as Neurobiotin [34], could conclusively determine if there is a presence or absence of gap junctions within the octopus intrinsic arm muscle.

Anatomical diversity within muscle layers may also contribute to improved arm control precision. No previous morphological or physiological distinctions have been made between the inner and outer layers of the longitudinal muscle of the cephalopod arm. Yet, we found a consistent distinction between these. From electron microscopy data, phasic contracting cephalopod muscle is characterized by a large central sarcoplasmic reticulum and mitochondrial core relative to the cell diameter (for review see [29]). In the SEM images of *O. bocki* (figures 5 and 6D; electronic supplementary material, figure S2), the fibres of the external longitudinal layer display a larger core and smaller cell diameter than the internal layer (figure 6E), suggesting the external layer is phasic-contracting while the internal is tonic-contracting. These morphological differences are only detectable in the SEM images of the *O. bocki*, which were taken from juvenile specimens from the tip of the arm. Therefore, it is possible that these differences are unique to *O. bocki* or to juvenile individuals. However, the fact that we have also found that the external longitudinal layer has significantly higher fibre density than the internal layer in adult *O. bimaculoides* and *A. aculeatus* gives us confidence that a similar anatomical inspection in these two species would also show morphological differences between these two layers. This significant muscle density difference between the internal and external longitudinal muscles, both within and among species, observed via considerable morphological sampling (six muscle blocks from 30 optical slices from two arms from two species; two *O. bimaculoides* and one *A. aculeatus*), suggests a shared functional origin that is important for arm muscle control.

A previous example of phasic and tonic-contracting physiologies (or, anaerobic versus aerobic) in different layers of the same muscle type occurs in the squid and cuttlefish mantle circular muscles [29]. The thick central zone of the mantle has few mitochondria and a poor vascular bed [35], while the thinner inner and external muscular zones have large mitochondrial cores, ample vascularization and higher oxidative enzyme activity [35,36]. The mitochondria-rich inner and outer zones are thought to be involved in the continuous, low-force contractions of the mantle in respiration and slow jetting, and therefore are probably aerobic and tonic-contracting fibres [29,37]. By contrast, the mitochondria-rich central zone is probably involved in fast jetting and exhibits anaerobic, phasic-contracting physiology [29,37]. It should be noted, however, that the radial muscles of the mantle are also integral to respiration, and these, incongruously, are mitochondria-poor [35]. However, in a separate example, the muscle of the cuttlefish tentacle has poor vascularization, suggesting anaerobic physiology, which aligns with the tentacles being used only intermittently during striking [38]. Extrapolating from these examples, it is possible to conclude that in the octopus arms, the thinner, mitochondria-rich external longitudinal layer primarily performs sustained postural contractions, while the thicker, mitochondria-poor internal longitudinal layer is engaged in more forceful, phasic movements. A peripheral position is more effective for bending [2,15]. Therefore, it is possible that the external longitudinal layer is preferentially used for sustained, structural bending contractions, while the internal layer is instead primarily used for fast shortening of the arm. It is unclear how the differences in muscle fibre density between layers found here may relate to these potential differences in function. It could be imagined that the higher density of the external layer allows for precise bending patterns along the length of the arm. However, future studies should further investigate the morphology, physiology and function of the longitudinal muscle to better clarify the differences between the internal and external layers.

## 4. Conclusion

Here, we confirm that each of the eight layers and the trabeculae of the intrinsic arm are composed of obliquely striated muscle fibres in three species of octopus: *O. bimaculoides*, *O. bocki* and *A. aculeatus*. We also found differences in muscle fibre morphology and density between the internal and external longitudinal muscle layers, positioned on either side of the median oblique muscle, suggesting a functional difference between the two. The octopus arm is a complex system, and new discoveries are continuously being made into its anatomy and functional control [24,25]. Future studies elucidating the functional role of each of these eight, or more, muscle layers are needed to determine the peripheral versus central control of arm dexterity.

## 5. Material and methods

### 5.1. Animals

All animal procedures were conducted in compliance with the U.S. Government Principles for the Utilization and Care of Vertebrate Animals Used in Testing, Research and Training, the EU Directive 2010/63/EU for cephalopod welfare [39,40], as well as ARRIVE guidelines regulating animal experimentation.

Adult *O. bimaculoides* specimens were collected off the coast of southern California and shipped overnight to Saint Paul, MN. Adult and juvenile *A. aculeatus* specimens were purchased from a commercial supplier (Corals Anonymous). Animals used for this study were size-matched as best as possible from those available (within approximately 30% of the mantle length of each other for each species). This resulted in female *O. bimaculoides* and male *A. aculeatus* being used for arm sampling. Octopuses were housed individually in 38 l glass aquaria enriched with clay-pot dens and artificial plants. Aquaria were supplied with a constant flow of filtered artificial seawater (Instant Ocean Salt Mix, purple box) at 16°C, 34 ppt salinity on average and a pH of approximately 8.3 was maintained in a light (red light) and dark cycle (12 h: 12 h). Red light is used to simulate low light conditions in their dens at more than 30 m depth, as the animals do not detect red wavelengths well, so it would appear dark to them. Upon arrival at the laboratory, octopuses were initially held in a quarantined, closed synthetic system that consisted of 250 l of recirculating artificial seawater for one week before being moved to a 1000 l recirculating artificial seawater system where 1.5% automatic water changes were performed twice per day. Both systems were equipped with mechanical and biological filtration. Water quality was monitored daily; ammonia and nitrite were 0 ppm, and nitrates ranged up to 10 ppm. Once in the laboratory, the animals were acclimatized for at least three weeks before being used for the experiment. Prior to the experiments, octopuses were fed three times per week under white light to simulate shallow water hunting with a mixture of food items, including pieces of thawed shrimp or live fiddler crabs. The *A. aculeatus* individual no. 2 was euthanized prior to growing to mature adult size, as it had stopped eating for unknown reasons.

### 5.2. Two-photon imaging of *Octopus bimaculoides* and *Abdopus aculeatus* muscle tissue

All animals were first anaesthetized in 110 mM magnesium chloride and 2% ethanol until movement of arms and mantle of the animal was unresponsive to touch and then euthanized in at least 4% ethanol (increased in 1% intervals until there was no movement in response to touch and it was consistent for at least 5 min). Animals were fixed in 4% PFA (Paraformaldehyde, Electron Microscopy Sciences, no. 15710) in fresh artificial seawater (Instant Ocean Salt Mix, purple box) with 10 times the volume of the animal at 21°C. *Octopus bimaculoides* individual no. 1 was placed in PFA for 48 h, while *O. bimaculoides* no. 2 was placed in PFA for 24 h, before both were moved to 1× PBS (Phosphate Buffered Saline, Sigma, no. P4417) and stored at approximately 4°C. Two *A. aculeatus* animals were used: one adult (no. 1) and one smaller individual (no. 2). *Abdopus aculeatus* no. 1 was fixed in PFA for 72 h, while *A. aculeatus* no. 2 was fixed for 48 h. Both were then stored in 1× PBS at approximately 4°C. For experimentation, the R2 and L2 arms were cut from the animals after sucker rows 7–10 where the webbing between arms ended, but were still close to the base of the arm. This position should avoid the documented autotomy zone observed in *A. aculeatus* [41]. The proximal 5–10 mm of the removed arm was then separated with a razor blade and cut to create a flat transverse surface (perpendicular to the long axis of the arm) for slicing. The skin, extrinsic arm muscles and sucker and sucker muscles were carefully removed using microscissors and blunt dissection. The tissue sample was then sliced in the cross-sectional orientation in PBS with a vibratome (Technical Products International, Classic 1000) into 100 μm thick axial slices perpendicular to the long axis of the arm. As the first several millimetres of the tissue block face were also discarded so that consistent slices were achieved, tissue sections were from sucker rows 8 to 11 across the arms sampled. The blade angle was set to 15°, the slicing speed set to 1.0 mm s^−1^ and the amplitude set to 1.0 mm. Slices were transferred to a glass microscopy slide using a 3 ml plastic transfer pipette with the tip removed to expand the opening. Excess PBS was wicked away with KimWipe tissues. Slices were incubated in 10 μl AlexaFluor 633 phalloidin dissolved in methanol (Invitrogen, no. A22284, 300 units ml^−1^), an F-actin fluorescent stain, on the slide for 5 min in the dark. Afterwards, excess dye was rinsed by applying approximately 1 ml of 1× PBS to the slice, which was wicked away with KimWipe tissues. Rinsing was repeated four times. A small drop of 80% glycerol was placed over the slice and gently stirred to encourage mixing. A coverslip (no. 1 thickness) was placed over the slide and held in place with a fishing weight. Nail varnish was applied to the edges of the coverslip to seal the sample and hold the coverslip in place. For each arm from each octopus, multiple vibratome axial sections were prepared and mounted as described above.

Prior to muscle scanning, selected images of a mounted whole slice were taken using a stereomicroscope (Leica M165FC, PlanApo 1× objective; Leica DFC310 FX colour camera). The mounted samples were imaged with a resonant scanning two-photon microscope (Bruker Ultima IV *in vivo*) using a Zeiss 25× objective (Carl Zeiss 440852-9870-000; 0.8 NA; 570 μm working distance; corrected to oil refractive index), a Newport Spectra-Physics InSight^®^ DS+^TM^ laser at 920 nm and an RFP (Red Fluorescent Protein, 610–650 nm) detection channel for AlexaFluor 633. The objective was immersed in 98% thiodiethanol. Images taken of the entire sample slice were digitally stitched together from tiled images spanning 448.5 μm^2^ (512 × 512 pixels, 0.876 × 0.876 μm pixel^−1^). To obtain larger images, with a more detailed field of view of the muscles so we could diagnose the presence of striations (or not) in every muscle layer (region), composite image stacks were stitched from images spanning 45.1 μm^2^ (512 × 512 pixels, 0.088 × 0.088 μm pixel^−1^). Multiple locations in the *x*–*y* plane were imaged with 20% spatial overlap to cover the structures of interest, and images were stitched together post hoc using the ImageJ (Fiji) plugin named ‘Stitching’ v.1.2 and the grid collection stitching option [42]. An automatic phase offset was applied, and three samples were taken per pixel. Volumetric scans were taken of each muscle group to detect striations, with stacks beginning at the surface of the tissue and penetrating at 1.0 μm step intervals in the *z*-axis to approximately 30 μm in depth. Images were saved in tiff format. If striations for each muscle layer could be repeatedly identified (i.e. greater than five examples of easily identified striated muscles, as marked in figures 3 and 4), only one vibratome section was surveyed and inspected for all 30 optical slices; otherwise, up to two more sections were surveyed using the same large area stitched z-stacks methods. The dimensions and area of the stitched tiled z-stacks in a 6 × 4 configuration were 270 × 180 μm (43 200 μm^2^). For visualization, the brightness of images of the entire slice was corrected linearly across the image, then adjusted with the ‘Auto’ algorithm within ImageJ (for figure 2; electronic supplementary material, figures S1, S3).

### 5.3. Scanning electron microscopy of *Octopus bocki* muscle tissue

SEM data were provided by the Crook laboratory of San Francisco State University. A full description of the methods and materials used to collect this data is provided in Neacsu & Crook [24]. Briefly, two subadult (mantle length 1.3–1.6 cm) *O. bocki* individuals were purchased from a commercial vendor (SeaDwelling Creatures, Los Angeles, USA). Animals were euthanized by immersion in isotonic MgCl2 (330 mM MgCl_2_.6H_2_O in RODI water, Reverse Osmosis De-Ionized) and 1% by volume ethanol, followed by decerebration. Small portions (1 mm in length) were removed from the distal ends of the L1 arm in animal no. 1 and R1 in animal no. 2. Samples were immediately fixed in 4% PFA and 2.5% glutaraldehyde in 0.1 M cacodylate buffer (pH 7.2) for 5–7 days at 4°C. Then, samples were shipped on ice to the 3DEM Ultrastructural Imaging and Computation Core at the Cleveland Clinic Lerner Research Institute (Cleveland, OH, USA) and postfixed with OsO4, dehydrated through a graded ethanol series, stained en bloc with uranyl acetate and embedded in epoxy resin for sectioning. Samples were cut and imaged using a Teneo Volumescope system (Thermo Fisher Scientific) and a Zeiss Sigma variable pressure field emission scanning electron microscope system (VP-FE SEM, Carl Zeiss Microscopy GmbH) equipped with a Gatan 3View in-chamber ultramicrotome stage. Both imaging stacks were collected in the cross-sectional (axial) orientation, perpendicular to the long axis of the arm. The sample from *O. bocki* no. 1 was imaged at 0.030 × 0.030 × 1.0 μm voxel^−1^, while the sample from *O. bocki* no. 2 was imaged at 0.050 × 0.050 × 1.0 μm voxel^−1^ part of the way through the scan and 0.080 × 0.080 × 1.0 μm pixel^−1^ through the remainder. During post-processing, images were converted from dm3 to tiff format, and contrast was automatically enhanced.

### 5.4. Calculation of muscle fibre density of internal and external longitudinal muscle

The density of muscle fibres within the internal (proximal to the median oblique muscle layer) and the external (distal to the median oblique muscle layer) layers was calculated from multiple locations (*n* = 6 for all slices, except *n* = 7 in R2 of *O. bimaculoides* individual no. 2) in both the R2 and L2 arms of three individuals (*O. bimaculoides* individuals no. 1 and 2, *A. aculeatus* individual no. 1). *Abdopus aculeatus* individual no. 2 was not included, as its muscular morphology shows very thin muscle layers in general and sparse longitudinal muscle, probably because of its juvenile status. Images of slices (0.876 μm^2^ pixel^−1^ resolution) were processed with a spatial high pass filter (threshold >40 pixels) to reduce irregularities in imaging (i.e. remove any very tiny shapes that are not muscle fibres such as tiny islands of tissue or lines from imaging), then processed with a maximum filter (radius two pixels) to remove fibre nuclei and mitochondrial cores. The six locations were chosen using anatomical markers that are consistent across all slices (electronic supplementary material, figure S3): markers were placed at the most oral and aboral points (oral and non-oral side of the animal) where the transverse and internal oblique muscle meet (denoted by stars). Aboral–oral running lines were drawn between each unilateral pair of these points. These lines were bisected twice to create three centroids per side (denoted by circles). The intact segment of internal longitudinal muscle (bounded by trabeculae) closest to each centroid was selected. Then, the external longitudinal segment corresponding to each internal longitudinal section was chosen. If there was any ambiguity about which segment should be chosen, the more aboral segment was used. Regions of interest were outlined manually in ImageJ (FIJI), keeping approximately two muscle fibre widths inside the edges of the muscle segment, to ensure that no trabeculae were included. Cell fibre density was calculated as the percentage of pixels within each segment falling above the mean of the high-pass-filtered image relative to the overall area of the segment. Statistics were performed using a one-sample Kolmogorov–Smirnov test (*p* < 0.05) to confirm the data were normally distributed and an N-way nested ANOVA (individual nested inside species).

### 5.5. Measurement of fibre and core area for internal and external longitudinal muscles

We first identified the boundaries of the median oblique and longitudinal muscle layers from the *O. bocki* SEM images from animals no. 1 and no. 2. Only one slice of the serial SEM stack was measured, as other slices would probably be pseudoreplication (as the muscles are long). From these representative images (cropped version in figure 5; electronic supplementary material, figure S2), we traced the perimeters of muscle fibre and mitochondria core from 10 randomly identified internal and external longitudinal muscles where the core was easily traced (some were not clear in the imaging). Tracing was outlined manually in ImageJ (FIJI), and areas were saved for analysis. Statistics were performed using a one-sample Kolmogorov–Smirnov test (*p* < 0.05) to confirm that the data were normally distributed and an N-way nested ANOVA.

## Data Availability

All analysed data reported in the figures can be located in Dryad [43]. This article does not report original code. All raw imaging data (approx. 1 TB) are available from the lead contact upon request. Supplementary material is available online [44].
